# *Salmonella*-Based Targeted Cancer Therapy: Updates on A Promising and Innovative Tumor Immunotherapeutic Strategy

**DOI:** 10.3390/biomedicines7020036

**Published:** 2019-05-02

**Authors:** Christian Ronquillo Pangilinan, Che-Hsin Lee

**Affiliations:** 1 Department of Biological Sciences, National Sun Yat-sen University, Kaohsiung 80424, Taiwan; chtianbiol@gmail.com; 2 Department of Medical Research, China Medical University Hospital, China Medical University, Taichung 404, Taiwan

**Keywords:** *Salmonella*, cancer treatment, targeted therapy, tumor immunotherapy

## Abstract

Presently, cancer is one of the leading causes of death in the world, primarily due to tumor heterogeneity associated with high-grade malignancy. Tumor heterogeneity poses a tremendous challenge, especially with the emergence of resistance not only to chemo- and radiation- therapies, but also to immunotherapy using monoclonal antibodies. The use of *Salmonella*, as a highly selective and penetrative antitumor agent, has shown convincing results, thus meriting further investigation. In this review, the mechanisms used by *Salmonella* in combating cancer are carefully explained. In essence, *Salmonella* overcomes the suppressive nature of the tumor microenvironment and coaxes the activation of tumor-specific immune cells to induce cell death by apoptosis and autophagy. Furthermore, *Salmonella* treatment suppresses tumor aggressive behavior via inhibition of angiogenesis and delay of metastatic activity. Thus, harnessing the natural potential of *Salmonella* in eliminating tumors will provide an avenue for the development of a promising micro-based therapeutic agent that could be further enhanced to address a wide range of tumor types.

## 1. Introduction

Bacteria-mediated tumor therapy has become one of the focal points in cancer therapeutic research over the past decades. The use of bacteria in cancer treatment may be favored over conventional treatment modalities such as surgery, chemo- and radiation therapies for several reasons [1]. First, surgery is only efficient in removing non-metastatic solid tumors, while bacteria harbor the potential to prevent metastasis. Second, tumor heterogeneity provides an avenue for the emergence of drug resistant and aggressive phenotypes whereas certain strains of bacteria used in tumor therapy can re-sensitize chemo-resistant tumor cells [1,2,3]. Lastly, the tumor microenvironment, such as the oxygen-depleted region of tumor, limits the effect of radiation therapy, whereas bacteria are able to disperse even at the hypoxic core [1,4].

One of the species used in bacteria-mediated tumor therapy is the gram negative, facultatively anaerobic *Salmonella* [5]. *Salmonella* sp. are able to penetrate and favorably invade tumor tissue by being attracted to the compounds produced by cancer cells as well as preferentially grow within tumor tissues [6]. Attenuated and engineered *Salmonella* have been demonstrated, both in in-vitro and murine models, to eliminate virulence while prompting antitumor activity via different mechanisms, largely due to the activation of tumor-specific humoral and cellular immune responses [7]. Despite being highly selective in targeting tumorigenic sites, the use of *Salmonella* in inhibiting tumor progression has encountered drawbacks such as host immunity against *Salmonella* antigens [8]. Interestingly, this problem has been addressed by modifications and alterations of the surface antigens or immunogenic components of *Salmonella* [9] and by coating the bacteria itself with certain materials like polymer to prevent the binding of *Salmonella*-specific antibodies, thus bypassing immune surveillance [10]. This review carefully explains the mechanisms underlying the tumor-targeting ability of *Salmonella* and its strategic activity in controlling tumor-specific responses that ultimately leads to inhibition of tumor growth and progression.

## 2. *Salmonella* Efficiently Targets and Invades Tumor

An interesting fact regarding *Salmonella* is their efficiency in selectively colonizing tumor tissue by being able to sense tumor-specific microenvironment. Solid tumors are characterized by depleted oxygen concentration ranging between 10 to 30 mmHg—otherwise known as hypoxia within the tumor tissue [11,12]—that is beneficial for *Salmonella* due to their facultative nature. To leverage this capability in targeting tumor tissue, an obligate anaerobic *S. typhimurium* strain (YB1) was generated by placing *asd* (aspartate-semialdehyde dehydrogenase) gene under the control of a hypoxia-conditioned promoter, that will be essential for survival only in a hypoxic condition [13]. Moreover, certain chemicals present in the tumor microenvironment, mostly in the necrotic region, could act as chemotactic agents that attract *Salmonella* to invade tumor tissue [6]. *Salmonella* have the ability to exploit ethanolamine as a signal to target and recognize niches including tumors [14,15]. The ability of *Salmonella* to efficiently reach and colonize tumor is facilitated by its motility, which is essential for effective dispersal in tumor [16]. Furthermore, *Salmonella* migration in the tumor is directed towards the core of the tumor and away from the vasculature [17]. Most *Salmonella* strains used for tumor therapy experiments and in clinical trials were attenuated, such as in the case of VNP20009 strain having partial deletion of the *msbB* gene for lipid A modification; in addition to that, the strain used bears *CheY* gene non-synonymous single nucleotide polymorphism, resulting in a reduced chemotactic ability [18,19]. Lipid A modification by *msbB* gene mutation reduced the lipopolysaccharide-associated toxicity or septic shock induction [20]. *CheY* gene is part of the two-component system CheA/CheY that is necessary or chemotactic response or motility and bacterial distribution in tumor tissue [6,18]; however, some reports showed that CheY protein deficiency does not compromise colonization in tumor [15]. In VNP20009, the efficiency of chemotactic response was only recovered after the replacement of the mutated *CheY* with the wild-type copy and restoring *msbB* gene [18,20] and further developed to enhance its hypoxia-targeting capacity [21]. Although several avirulent *Salmonella* strains generated via mutation demonstrated a reduced targeting and fitness ability, a variety of mutants categorized as class 1 such as *htrA*, *SPI*-2, and *STM3120* mutations did not exhibit significant change in fitness and exclusive targeting of tumors [22]. In vitro targeting using tumor-on-a-chip device and in vivo targeting in tumor-bearing mice was achieved by Trg-deficient *Salmonella* through penetration of, and dispersal in, the quiescent tumor region [23]. 

## 3. Modified *Salmonella* Bypasses Antibacterial Immune Response

One of the drawbacks of using bacteria as an antitumor agent is the host’s immune response, triggered upon an increase in bacterial concentration, and ultimately leading to clearance of the introduced bacteria [24]. In fact, preexposure to *Salmonella* may hinder the therapeutic potential of bacteria-mediated tumor therapy [8,25]. This limiting effect of preexposure has been addressed by engineering *Salmonella* strain SF200 resulting to a modified Lipid A structure via *∆lpxR9 ∆pagL7* and *∆pagP8* deletions, and *∆ydiV* and *∆fliF* mutations to modify flagella synthesis [9,25]. The optimized SF200 strain showed significantly higher tumor regression in naïve and immunized tumor-bearing mice as compared to non-optimized variant, SL7207 that showed relatively low tumor regression [25]. Moreover, SF200 induced cytokine levels in immunized mice comparable to naïve mice, while SL7207 showed significantly lower cytokine levels in tumors of immunized mice compared to naïve mice [25]. In another experiment, *Salmonella* was encapsulated with poly(allylamine hydrochloride) or PAH to prevent binding of antibodies specific for *Salmonella* [10]. PAH-coated *Salmonella* (PAH-S.C.) did not elicit significant change in its tumor targeting ability and effectively cleared tumor in naïve and immunized tumor-bearing mice involving the recruitment of infiltrating immune cells such as neutrophils and macrophages [10]. 

## 4. *Salmonella* Promotes Activation of Antitumor Immunity 

**Figure 1:**
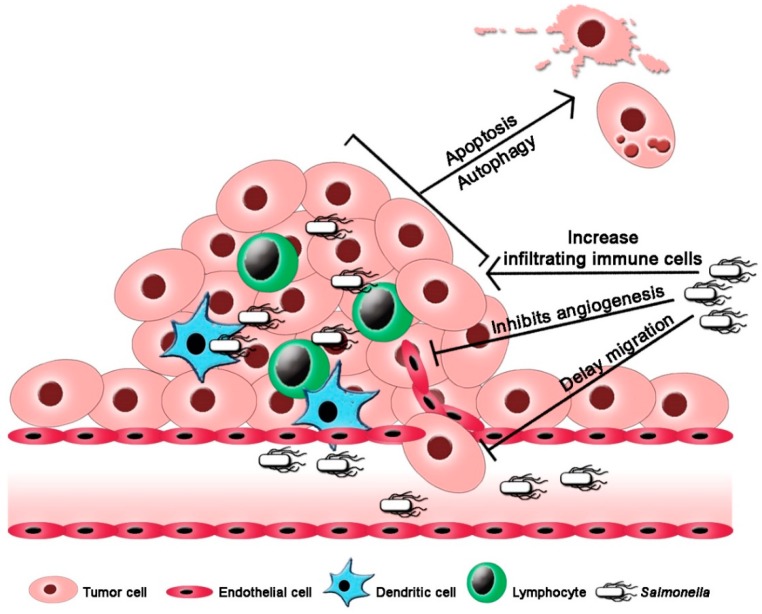
*Salmonella*-mediated tumor immunotherapy. *Salmonella* triggers immune cells infiltration into tumor tissue and coax tumor cells self-destruction while preventing tumor microvascularization and delaying tumor cell migration.

The development of various immune evading strategies by tumor cells enabled the generation of more aggressive phenotypes [26]. These highly aggressive phenotypes have led to the establishment of resistance to tumor immune therapy [26,27]. Systemic administration of *Salmonella*, shown in Figure 1, triggers immune cell infiltration and induction of proinflammatory cytokine expressions such as IL-1β and TNF-α [5,28]. Also, macrophages in *Salmonella*-colonized tumor express and activate the inflammasome pathway involving NLRP3, IPAF and caspase-1 p10 [29]. When *Salmonella* gained access to the tumor microenvironment, intratumoral CD11b^+^ myeloid cells accumulate and are coaxed to undergo phenotypic and functional maturation, impairing their suppressive activity [30,31]. The decrease in suppressive activity after *Salmonella* treatment involves downregulation of immunosuppressive factors, particularly arginase-1, IL-4, TGF-β, and VEGF, and an enhanced expression of inducible nitric oxide synthase (iNOS) and IFN-γ [31]. The enhanced expressions of iNOS, IFN-γ and other IFN-inducible chemokines may play a crucial role in the recruitment of neutrophils, activated CD8+ T cells and an increase in intratumoral activated NK cells [31,32,33]. It was previously reported that TLR4 signaling is involved in the *Salmonella*-induced cytokine expression [32] and that the lipopolysaccharide (LPS) of *Salmonella* might be crucial in the activation and recruitment of immune cells and the subsequent production primarily of TNF-α [34]. In a recent study, an engineered *S. typhimurium* strain secreting heterologous flagellin B (FlaB) in tumor tissues first induced the recruitment of infiltrating immune cells via TLR4 signaling followed by the activation of the recruited intratumoral macrophages via TLR5 signaling which appears to have caused the increase in the synthesis of cytotoxic mediators and cytokines [35]. In another study, the flagellin of *S. typhimurium* fused with peptide P10 of the gp43 protein from *Paracoccidioides brasiliensis* activated TLR5 signaling that helped impair the metastatic activity of melanoma in vivo [36]. 

Bacterial LPS and the enhanced synthesis of TNF-α induced the activation of CD8+ T cell that plays a major role in tumor regression [34], consistent with the findings that the antitumor activity of host immune system involves both CD4+ and CD8+ T cells rather than solely relying on innate mechanisms [37,38]. Lysis of tumor cells by anti-*Salmonella*-specific T cells further recruits infiltration of CD8+ T in *Salmonella*-colonized tumor that might eventually result in the uptake of tumor debris by antigen-presenting cells leading to presentation to naïve T cells and activation of tumor-specific T cells [7,37,39]. Previous reports have already pointed out that bacteria-induced gap junctions, such as connexin-43 (Cx43), can be formed between tumor and dendritic cells and promote tumor antigen cross-presentation [40]. The upregulation of Cx43 in tumor after *Salmonella* treatment explains tumor antigen cross-presentation leading to immune cell-mediated antitumor activity [41].

The activation of antitumor immunity mediated by *Salmonella* treatment does not rely only on the recruitment of a variety of infiltrating immune cells as mentioned previously. Interestingly, *Salmonella* invasion of tumor tissue interferes with the immunosuppressive nature of tumor microenvironment in a variety of mechanisms [5,7]. The immunosuppressive factors arginase-1, IL-4, TGF-β, and VEGF were known to be upregulated in many solid tumors but were found to be downregulated following *Salmonella* treatment [31]. Another immunosuppressive factor, the indoleamine 2, 3-dioxygenase 1 (IDO), is involved in mediating activation of regulatory T cells by increasing kynurenine concentration [42]. In a recent study, *Salmonella* downregulated IDO expression in B16F10 and 4T1 tumor cells via inhibition of AKT /mTOR/p70S6K signaling pathway, and thus resulted in a decrease in kynurenine concentration [43]. Since kynurenine is involved in competent T cell apoptosis [44] and *Salmonella* can reduce kynurenine, T cell survival increased as seen in Jurkat cells (T cells) cell viability cultured in a medium of tumor cells previously treated with highest dose of *Salmonella* [43]. In another study, *S. typhimurium* was transformed with shRNA targeting IDO to further enhance intratumoral cell death by inducing an increase in polymorphonuclear neutrophils (PMN) activity [45].

## 5. *Salmonella* Mediates Tumor Cell Self-Destruction

Programmed cell death, and apoptosis in particular, is crucial in tumor clearance involving the activation of caspase cascade in response to cancer therapy; however, in a state of high-grade malignancy, apoptosis remains attenuated [2]. The efficiency of *Salmonella* in combating tumor malignancy primarily involves the activation of cell death pathways (Figure 1 and Table 1) by nutrient competition and an enhanced stimulation of tumor-specific immune responses [5,17]. To highlight the mechanism, *Salmonella* treatment increased the expression of cleaved caspase-3 required in the activation of apoptosis via caspase cascade system in two melanoma models, K1735 and B16F10 [46]. Apoptotic response of tumor was only reversed in pan-caspase inhibitor Z-VAD-FMK-treated tumor cells which verified the theory that apoptosis is involved in *Salmonella*-mediated tumor cell death [46]. Furthermore, autophagy adds up to the layer of responses following *Salmonella* treatment as indicated by the upregulation of Beclin-1 along with an enhanced conversion of LC3-I to LC3-II, an autophagosomal marker [46]. The activation of autophagic signaling pathway is mediated by downregulation of AKT/mTOR/p70S6K being a crucial target of *Salmonella* in tumor cells [46]. In another study, *Salmonella* was used as a vector to carry second mitochondria-derived activator of caspases (Smac) and tumor necrosis factor-related apoptosis-inducing ligand (TRAIL) genes which enhance apoptotic cell death and resulted to almost 90% regression of tumor growth in murine melanoma, lung carcinoma, and mammary carcinoma models [47,48]. 

## 6. *Salmonella* Diminishes Tumor Metastasis 

The prevention of metastatic activity by *Salmonella* through various mechanisms prevent tumor cells from migrating (Figure 1 and Table 1). Failure to detect the advancement of tumor into metastatic state where cancer cells gain access to different sites of the body leads to a significantly poor prognosis which, in turn, dramatically affects patients’ survival. This is primarily due to the changes in the behavior of cells in the primary tumor causing migration and aggressive invasion of the proximal and even distal organs forming secondary lesions [52,53].

The initiation of angiogenic sprouting in tumor marks the dramatic shift from dormancy to tumor progression [2,54]. Angiogenesis is not only critical to tumor growth per se, but also to tumor migration because the new blood vessels formed provide the major route used by disintegrating tumor cells to escape from the primary tumor and start migration [55]. Many studies have already demonstrated the role of this angiogenesis in tumor metastasis and, thus, that the inhibition of angiogenic sprouting may help contain the tumor and stop its metastatic activity. The regulation of angiogenic sprouting is orchestrated by the angiogenic factors *vascular endothelial growth factor-A* (VEGF-A) and thrombospondin-1 (TSP-1) that either stimulates or inhibits cell-surface receptors, respectively [56]. In the tumor microenvironment, the presence of oncogenic signals and hypoxic condition activate certain transcription regulators such as *hypoxia-inducible factor 1-alpha* (HIF-1α) to induce upregulation of VEGF [57,58,59] that largely influences tumor vascularization. In a study by Tu et al. [49], *Salmonella* inhibited angiogenesis by negatively regulating VEGF expressions in two tumor cells namely, B16F10 and 4T1. The downregulation of VEGF is attributed to the downregulation of its gene regulator HIF-1α, suggesting that the anti-angiogenic activity of *Salmonella* targets the HIF-1α dependent pathway. The activation of HIF-1α is mediated by AKT/mTOR/p70S6K cascade [49,59,60,61] which was found to be significantly reduced after *Salmonella* treatment as indicated by decline in AKT, mTOR and p70S6K phosphorylation. Interestingly, the frequency of viable human microvascular endothelial cells (HMEC-1) declined significantly post-treatment with conditioned medium of either *Salmonella*-treated B16F10 or 4T1 cells [49]. Consistent with the findings already mentioned, in vivo study using tumor-bearing mice showed reduced VEGF levels in tumor tissue and reduced tumor microvessel density that is necessary to confine tumor in the primary site [49]. 

For metastatic tumor to escape primary site and eventually migrate to other organs to seed secondary tumors, metastatic tumor cells rely on an important process involving proteolytic degradation of extracellular matrix (ECM) by proteinases such as matrix metalloproteinase [62]. Matrix metalloproteinase, a member of the zinc-dependent endopeptidases family, is known to modulate ECM remodeling in normal development, inflammation, and wound healing [63,64,65,66]. In various types of tumors, MMP-9 expression is primarily implicated in metastatic phenotypes by acting upon the ECM components thereby altering adhesive capability and in promoting tumor vascularization [67,68,69]. MMP-9 expression contributes to the epithelium to mesenchymal transition (EMT), serving as one of the markers of cancer stem cells [70]. It is therefore advantageous to utilize therapeutic strategies targeting MMP-9 expression. Negative regulation of MMP-9 expression has also been demonstrated to suppress metastatic activity of prostate cancer [71,72] malignant glioma [73] and renal cell carcinoma [74]. When *Salmonella* is used as an antitumor agent, it inhibits not only tumor vascularization [49], but also prevents EMT by keeping an intact ECM via suppression of MMP-9 expressions [50]. The inhibition of tumor cell migration observed in wound-healing and transwell assay was found to be due to MMP-9 suppression that is controlled by AKT/mTOR axis. The downregulation of phospho-AKT/phospho-mTOR after *Salmonella* treatment in both B16F10 and LL2 cells resulted in a decrease of MMP-9 expression which was reversed in tumor cells transfected with constitutively active AKT [50]. 

## 7. *Salmonella* Enhances Chemosensitivity of Tumor

**Figure 2:**
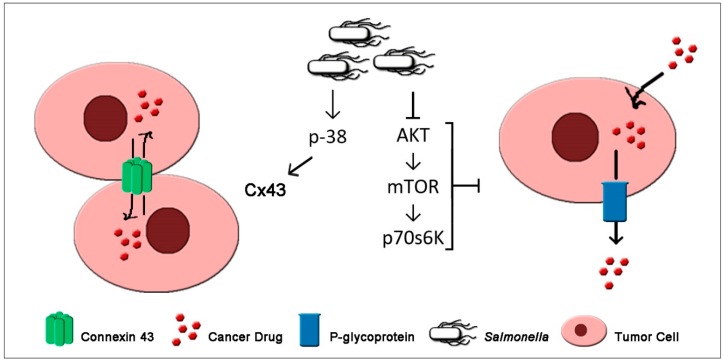
*Salmonella* enhances gap intercellular communication (GJIC) via gap junctions and alters membrane permeability to re-sensitize drug-resistant tumor cells.

An alarming increase of chemoresistance fueled by tumor heterogeneity among genetically-distinct sub-populations of tumor cells [3] demanded the exploration of alternative efficient therapeutic strategies that could combat drug resistance or a way to sensitize an already resistant phenotype. Mechanism of multi-drug resistance among various types of cancer primarily includes alteration of membrane permeability via transporter proteins, i.e. P-glycoprotein (P-gp), involved in drug efflux [75,76]. Many other resistance mechanisms causing poor responses to various drugs include alteration of target enzymes, alteration of drug metabolism, suppression of apoptosis, and enhancement of DNA repair mechanisms, among others [76,77]. Surprisingly, as shown in Figure 2 and Table 1, *Salmonella* can sensitize drug resistant-tumor cells (K1735 melanoma cells) to cisplatin—a cytotoxic drug—by enhancing gap intercellular communication (GJIC) mediated by the upregulation of connexin 43 (Cx43) [41]. Furthermore, the study showed that Cx43 was mediated by p38 signaling cascade as determined by using inhibitor of p38 that blocked the *Salmonella*-mediated Cx43 expression. Furthermore, overexpression Cx43 increased the expression of p38, while Cx43-silenced cells showed no significant changes on p38 expressions [41]. A recent study revealed that the same *Salmonella* mediated Cx43 upregulation and also contributed to the downregulation of P-gp, further confirming the ability of *Salmonella* to sensitize multidrug-resistant tumor cells [51]. Previous findings have also demonstrated the potential of *S. enterica* serovar Typhimurium in modulating the expression of P-gp in the epithelial lining of the intestine [78]. Poor prognosis in many patients with solid tumors and blood malignancies often have unfavorably enhanced P-gp efflux potential that drives cancer drug out of tumor cells [76]. The expression of P-gp, which is normally controlled by AKT/mTOR signaling via p70s6K phosphorylation [79,80], was significantly reduced in B16F10 and 4T1 cells after *Salmonella* treatment in a dose-dependent manner [51]. The downregulation of P-gp mediated by *Salmonella* showed a significant increase in Rho-123 intracellular accumulation suggesting suppressed P-gp transport activity, and an increased susceptibility of tumor cells to 5-FU [51]. Previously, Mercado-Lubo et al. [81], reported that SipA, a type III secretion effector in *S. enterica*, controls P-gp levels involving caspase-3-mediated protein degradation rather than transcriptional control. 

## 8. Combination Therapy with *Salmonella* Further Improves Tumor-Regression 

In recent years, bacteria-mediated tumor therapy is no longer centered on using *Salmonella* alone as a means of combating cancer. An increasing number of studies have now been reported to maximally exploit the potential of *Salmonella* in combination with other therapeutic strategies to combat cancer [1,7]. 

The ability of *Salmonella* to sensitize multi-drug resistant tumors have encouraged researchers to use various cancer drugs such as cisplatin or 5- Fluorouracil combined with *Salmonella* treatment [41,51]. A combined *Salmonella* therapy and cyclophosphamide drug treatment improves tumor regression and significantly decreased tumor micro-vascularization in melanoma model [82]. Recently, Bascuas et al. [83] demonstrated that *Salmonella* treatment in B-cell non-Hodgkin lymphoma (B-NHL)-bearing mice enhanced the effect of chemotherapy using CHOP (cyclophosphamide, doxorubicin, vincristine, and prednisone). CHOP treatment in mice model prior to *Salmonella* treatment revealed enhanced NK cell cytotoxic activity and a significantly higher lymphoma-specific humoral and cellular immune responses compared to *Salmonella* alone nor CHOP alone treatment [83]. 

In another study, adoptive T cell therapy was combined with *Salmonella* treatment, in which intravenous injection of either viable or heat killed (HK) *Salmonella* enhanced the proliferation of adoptively transferred OT-1 T cells and significantly improved tumor regression compared to OT-1 T cells alone [84]. Despite the successful regression of tumor by HK *Salmonella* combined with adoptive T cell therapy, one mouse died after 16 days post-treatment indicative of potential increase in toxicity. The observed mortality may be due to elevated levels of IL-6 and other pro-inflammatory cytokines which can be addressed by applying neutralizing antibodies [84]. 

The hypoxia-targeting ability of *Salmonella* was used in tandem with photothermal therapy using the carbon-based nanomaterial, polydopamine [85]. In the study, *Salmonella* strain VNP20009 was coated with polydopamine, designated as pDA-VNP, to act as photothermal agent. The efficacy of photothermal therapy is mediated by photothermal agents, such as pDA, to induce elevation of local temperature by converting incident light into heat [86]. The findings suggest that the targeting ability of *Salmonella* successfully delivered polydopamine to the tumor site and that near-infrared irradiation induced significant increase in temperature in the tumor site enough to cause tumor cell decay. In vitro cytotoxicity revealed significantly lower viable B16F10 cells in pDA-VNP post-irradiation group compared with VNP20009 alone [85]. Similar findings were observed in melanoma mouse model as indicated by apoptotic and necrotic cells and confirmed by infrared thermal imaging of mice showing significant increase of temperature in pDA-VNP with irradiation [85]. 

## 9. Clinical Trials, Challenges and Future Perspectives

The first attempt of using *Salmonella* in preclinical studies and clinical trials for the treatment of advanced or metastatic tumor can be dated back in the early 2000 where, in particular, the strain VNP20009 described earlier in this paper, was developed by Vion Pharmaceutics Inc [87]. Unfortunately, the phase 1 clinical trials of VNP20009 was discontinued due to low tumor regression and incidence of side effects at high dosage [85,87]. Despite the outcome, VNP20009 has been safely administered via intravenous infusion resulting in increased circulation of proinflammatory cytokines and marked colonization in the tumor biopsies of three out of 25 patients in the trial [87,88]; this means, it is safe to assume that *Salmonella* can be further developed to improve the outcomes of future clinical trials. One hindrance affecting the tumor targeting ability of VNP20009, as reflected in the results of phase-1 clinical trials, is the point mutation in *CheY* gene that forms part of the two-component system CheA/CheY associated with motility and bacterial distribution of *Salmonella* in tumor tissue [6,18]. The chemotactic ability of VNP20009 was enhanced, by 69% efficiency, with respect to the parental strain, after replacing the *CheY* mutated copy with the wild-type sequence [18] and then optimized by restoring *msbB* gene in VNP20009 *CheY*^+^ that further increased chemotactic mobility [20]. Another strategery that may better equip *Salmonella* in targeting tumor sites is surface modification. Recently, Park et al. [89] modified the *S. typhimurium* to display arginine-glycine-aspartate (RGD) peptide sequence on the external loop of outer membrane protein A (OmpA). RGD peptide binds efficiently to αvβ3 which is overexpressed in most tumor cells; therefore, RGD-displaying *Salmonella* can target tumor overexpressing αvβ3 at high efficiency while weakly binding to αvβ3-negative cells [89].

Majority of *Salmonella* strains used as anticancer agents were developed by disabling genes associated with virulence which may be required for immune activation associated with antitumor response, an essential aspect that makes cancer immunotherapy efficient. Several reports have already provided solutions for the improvement of the antitumor potential of *Salmonella*, mostly by surface modification and/or engineering of various strains, such as VNP20009, A1-R and the avirulent ∆ppGpp, among others, but not directly addressing over-attenuation [5,90]. Low tumor regression possibly due to over-attenuation can be addressed by altering a virulence factor to be expressed under an inducible promoter such as PBAD requiring arabinose as an inducer in an in vitro system [91]. The concentration of arabinose becomes heavily diluted after administration, which will eventually lead to a halted virulence factor expression. In turn, *Salmonella* will become attenuated after a few rounds of replication without the inducer arabinose. For instance, the transcriptional regulator *phoP* that is responsible for *Salmonella* virulence and a regulator of LPS structural modifications, has been engineered to be expressed under PBAD promoter [92]. By so doing, a more vigorous anti-tumor activity may be prompted compared to an over-attenuated strain. This opens another area of investigation in cancer therapeutics that is focused on using anticancer *Salmonella*.

In another phase 1 clinical trial, *S. typhi* Ty21a was used as a vector to deliver the oral DNA vaccine VXM01 targeting the vascular endothelial growth factor receptor 2 (VEGFR-2) as an antiangiogenic intervention for advanced pancreatic cancer patients [93,94]. Preliminary findings suggest that Ty21a can be safely administered with only minimal adverse effect, relatively substantial tumor regression and a significant reduction of tumor perfusion along with elevated levels of serum anti-angiogenic biomarkers [94]. In the phase 1 trial extension, treatment-associated adverse reactions were significantly decreased along with increased vaccine specific T cell responses [95]. Another vaccine strain of *Salmonella* called TXSVN is set for a phase-1 clinical trial for multiple myeloma [96]. This genetically altered *Salmonella* produces tumor-associated antigens (TAAs) known as Survivin and is expected to induce tumor-specific immune response.

Ultimately, with the right attenuated strain that is safe for systemic administration harboring the essential elements involved in tumor targeting, as well as efficiently prompting oncolysis, *Salmonella*-based targeted cancer therapy will play a significant part in cancer immunotherapy. In addition, investigating further on the innate mechanisms of *Salmonella* in disrupting tumor growth and progression may prove helpful in maximizing the potential of this bacteria for use in monotherapy, vaccine delivery vector or in tandem with other useful therapeutic interventions. 

## 10. Conclusions

The increasing number of relevant findings from cell-based to preclinical researches clearly suggest that *Salmonella*-based targeted therapy is a promising therapeutic candidate that could potentially become a mainstream therapeutic strategy in cancer treatment. Because *Salmonella* can efficiently work in a multifaceted interplay between upregulation of immunomodulatory molecules and downregulation of aggressive phenotype-related proteins to counteract various protumor cellular processes, it can be utilized to improve survival of cancer patients or can be enhanced to improve outcomes of existing treatment strategies. Most of the obstacles in using *Salmonella* have already been addressed or at least, lessened, such as potential toxicity and host immune response against the bacterial agent itself. *Salmonella* may not be the holy grail, in terms of cancer therapeutics, but further improvement of the treatment, through bioengineering and/or combinatorial treatments, may prove to be significantly effective in combating high-grade cancer malignancies.

## Figures and Tables

**Table 1:** A summary of the tumor cell signaling targets of *Salmonella*-mediated tumor immunotherapy.

Protein Targets	Control	Mechanisms	Ref.
Connexin 43	▲	Increases tumor cell chemosensitivity	[41]
IDO	▼	Suppresses tumor immune tolerance	[43,45]
Beclin-1 and LC3	▲	Induce autophagy	[46]
Cleaved Caspase-3	▲	Activates apoptosis	[46]
HIF-1/VEGF	▼	Inhibits angiogenesis	[49]
Matrix MMP-9	▼	Delays cell migration and metastasis	[50]
P-glycoprotein	▼	Increases tumor cell chemosensitivity	[51]

Note: ▲-Upregulation; ▼-downregulation.

## Abbreviations

AKT	protein kinase B
*asd*	aspartate-semialdehyde dehydrogenase gene
B-NHL	B-cell non-Hodgkin lymphoma
CHOP	cyclophosphamide, doxorubicin, vincristine, and prednisone
Cx43	connexin-43
ECM	extracellular matrix
EMT	epithelium to mesenchymal transition
GJIC	gap junction intercellular communication
HIF-1	hypoxia inducible factor-1
HK	heat killed
IDO	indoleamine 2, 3-dioxygenase 1
IFN-γ	interferon-γ
IL	interleukins
iNOS	inducible nitric oxide synthase
LPS	lipopolysaccharide
MMP	matrix metalloproteinase
mTOR	mammalian target of rapamycin
OmpA	outer membrane protein A
p70S6K	ribosomal protein S6 kinase beta-1
P-gp	P glycoprotein
PAH	poly(allylamine hydrochloride)
pDA	polydopamine
PMN	polymorphonuclear neutrophils
RGD	arginine-glycine-aspartate
shRNA	short hairpin ribonucleic acid
TGF-β	transforming growth factor-β
TLR	toll like receptor
TNF-α	tumor necrosis factor-α
TRAIL	tumor necrosis factor-related apoptosis-inducing ligand
TSP-1	thrombospondin-1
VEGF	vascular endothelial growth factor.
